# Correlation between skin patch testing and clinical outcome in total knee arthroplasty, a serial prospective study^☆^

**DOI:** 10.1016/j.abd.2022.04.007

**Published:** 2022-12-23

**Authors:** Maria Claudia Carvas Passarelli Tirico, Vitor Manuel da Silva Reis, Valeria Aoki, Marco Kawamura Demange, Luis Eduardo Passarelli Tirico

**Affiliations:** a Medical school, Hospital das Clínicas, Universidade de São Paulo, São Paulo, SP, Brazil; b Institute of Orthopedics and Traumatology, Hospital das Clínicas, Universidade de São Paulo, São Paulo, SP, Brazil

Dear Editor,

Metal Allergic Contact Dermatitis (ACD) has a wide distribution, in which the main allergens involved are nickel, chromium and cobalt.^1^ Contact of these substances with the skin of a previously sensitized individual triggers an inflammatory response that can be diagnosed through patch testing.^2^, ^3^

Conjectures have been made on whether it would be possible to develop an allergy to metals from implants such as plates, screws, and joint replacements,^4^, ^5^ and what could be its clinical repercussions. Would previously allergic patients have an increased risk of failure with a traditional implant? In addition, metallic components tend to be the main suspects; however, joint replacements often use cement composed of polymethylmethacrylate. There is substantial evidence of the allergenic characteristics of acrylates present in dental resin and gel nails.^6^, ^7^

The primary objective of this study was to assess the presence of allergic sensitization through patch testing after Total Knee Arthroplasty (TKA) and to assess a possible correlation between a positive patch test and clinical outcome. Finally, we authors intended to determine whether the test might influence the choice of implant.

Patients with inflammatory, autoimmune, or immunosuppressive diseases; systemic use of corticosteroids or immunosuppressants; use of topical corticosteroids at the application site; evidence of infection by the human immunodeficiency virus; the presence of atopic dermatitis; or presence of metallic synthesis materials due to orthopedic or bucomaxillofacial surgery were excluded.

Patients underwent a standardized contact test at time zero ‒ the day of admission for the surgery and after 6 weeks, 6 months, and 1 year. Tested substances were pure petrolatum jelly (control), Nickel sulfate 5% (Ni), potassium bichromate 0.5% (Cr), Cobalt chloride 1% (Co), Ethyl Acrylate 1% (EA), Ethylene Glycol Dimethacrylate 2% (EGDM) and 2-Hydroxyethyl Methacrylate 2% (2-HEM), all diluted in a petrolatum solution.

Fourteen patients aged between 54 and 85 years were included. There were 2 infectious complications, and 5 patients were unable to perform the 12-month test (Table 1).

**Table 1:** Demographic, clinical and surgery data from the patients submitted to total knee arthroplasty.

Number of patients	14
Minimum age (years)	54
Maximum age (years)	85
Female	9
Male	4
Past history of metal allergy	2
Complications (infection)	2
Loss of follow-up	5

Although only 2 patients reported previous allergies to metals, 7 patients were identified with a positive test for any metallic substance at time zero, that is, an initial incidence of 50%, higher than expected since there was no selection bias. This finding suggests that performing a preoperative contact test as a way of guiding the choice of implant, should not be restricted only to those who report previous allergies, as suggested by Schalock et al.^4^ In fact, those who report previous allergic symptoms are likely to be positive. On the other hand, it would be more interesting to evaluate patients who deny a previous history of allergy to metal or acrylates, as they may be sensitive to these substances but just not be aware of it.

Perhaps the most relevant observation of this study was the high conversion rate of the tests in relation to metals during the follow-up. The first test revealed that 50% of the patients had a previous sensitivity to any of the metals. At the end of the study, we observed that all patients were positive for at least one of the metals tested, with the majority being positive for more than one metal per test (Table 2).

**Table 2:** Positive patchtest results for metals per each patient submitted to total arthroplasty.

	Zero	6-Weeks	6-Months	12-Months
**P #1**	Cr / Co	Cr	Cr / Co	Cr
**P #2**			Ni / Cr / Co	
**P #3**			Cr / Co	Ni / Cr / Co
**P #4**	Ni	Ni / Cr / Co	Ni / Cr / Co	Ni / Cr / Co
**P #5**		Ni / Cr / Co		
**P #6**		Ni	Ni	
**P #7**	Ni	Ni / Cr / Co	Ni / Co	Ni / Co
**P #8**	Ni / Co	Ni / Cr / Co	Ni / Co	Ni / Co
**P #9**	Ni / Cr / Co	Ni / Cr / Co	Ni / Cr / Co	Ni / Cr / Co
**P #10**	Ni / Co		Ni / Cr	
**P #11**	Co		Ni / Co	
**P #12**		Ni / Cr / Co		
**P #13**			Co	
**P #14**		Co	Co	

Ni, Nickel; Cr, Chromium; Co, Cobalt.

EA was the substance with the highest positive index in the first test, in which 12 patients were reactive. During follow-up, only 1 patient developed a response to this allergen. EGDM had only 1 positive patient at time zero, but the reaction did not persist during follow-up, while another 3 patients became positive at 12 months of follow-up. No patients were positive for 2-HEM reaction at the initial test, but 2 patients developed a reaction to this compound afterward (Table 3).

**Table 3:** Positive patch test results for acrylates per each patient submitted to total arthroplasty.

	Zero	6 Weeks	6 Months	12 Months
**P #1**	EA		EA	EA
**P #2**				EA EGDM
**P #3**		EA		EA
**P #4**	EA	EA / EGDM	EA	EA / EGDM 2-HEM
**P #5**	EA	EA	EA	
**P #6**	EA	EA	EA	
**P #7**	EA	EA	EA	EA
**P #8**	EA / EGDM	EA	EA	EA
**P #9**	EA	EA / EGDM 2-HEM	EA	EA / EGDM
**P #10**	EA		EA	
**P #11**	EA	EA	EA	
**P #12**	EA		EA	
**P #13**	EA		EA	
**P #14**	EA	EA	EA	

EA, Ethyl Acrylate; EGDM, Ethylene Glycol Dimethacrylate; 2-HEM, 2-Hydroxyethyl Methacrylate.

We can, therefore, imagine that the presence of knee arthroplasty implants may in fact cause allergic sensitization. However, a correlation between test positivity and an unfavorable clinical outcome was not observed, since to date all patients have continued to be followed up for more than 5 years after surgery with good clinical outcomes. On average, 150 TKA are performed per year in service and to date, there have been no diagnosed cases of allergy. Therefore, we must carefully analyze the proposal by Krecisz et al.,^8^ who suggest that all patients who have confirmed allergies should receive implants free of these substances. The scientific evidence behind this statement is still controversial and could generate excessive costs to the health system since special hypoallergenic implants are more expensive than regular implants.^8^, ^9^

Unfortunately, the present analysis was based on a number of patients smaller than desired, and thus, we did not obtain sufficient statistical power to scientifically support the our hypotheses.

The presence of allergies to orthopedic implants is still very controversial. There is great difficulty in conducting adequate studies to assess this topic, as a large number of participants and long-term follow-up are required. The use of the patch test for this purpose is an interesting tool because it is noninvasive and a well-established test in dermatology.

This study allowed us to determine that the presence of allergic sensitization after TKA is a likely phenomenon. However, it does not seem to be related to a negative clinical outcome. In addition, we were able to conclude that preoperative patch testing would be interesting for patients who deny the previous allergy to metals or acrylates, as it is possible that they have not yet manifested signs of dermatitis. Finally, the evidence is insufficient for this test to be routinely used to guide the choice of implant.

## IRB approval statement

This study was approved by the scientific commission of our service (CAPPESQ 13079 – IOT 1105) and registered in the *Plataforma Brasil* (CAAE 45398015.0.0000.0068).

## Financial support

None declared.

## Authors’ contributions

Maria Claudia Carvas Passarelli Tirico: Statistical analysis; study design and planning; preparation and writing of the manuscript; obtaining, analyzing and interpreting data; intellectual participation in propaedeutic and/or therapeutic conduct of cases studied; critical review of the literature.

Vitor Manuel da Silva Reis: Final version approval; study design and planning; critical review of the manuscript.

Valeria Aoki: Final version approval; study design and planning; critical review of the manuscript.

Marco Kawamura Demange: Final version approval; critical review of the manuscript.

Luis Eduardo Passarelli Tirico: Final version approval; study design and planning; preparation and writing of the manuscript; obtaining, analyzing and interpreting data; effective participation in research guidance; critical review of the manuscript.

## Conflicts of interest

None declared.
